# Comparative study of treatment adherence, treatment-related attitudes, and background factors in schizophrenia spectrum and bipolar patients

**DOI:** 10.1192/j.eurpsy.2022.1942

**Published:** 2022-09-01

**Authors:** H. Janicsák, Z. Grenda, D. Dudas, A. Soos, M. Sztyehlik, G. Gazdag

**Affiliations:** Jahn Ferenc South-pest Hospital, Centre Of Psychiatry, Budapest, Hungary

**Keywords:** bipolar disorder, medication adherence, schizophrénia

## Abstract

**Introduction:**

Previous research confirmed high rates (20-89%) of non-adherence to medication among psychotic and bipolar patients. Results suggests that positive attitude to treatment has the highest influence on patients’ adherence and significant differences between treatment related attitudes and treatment adherence of psychotic and bipolar patients were found.

**Objectives:**

The aims were to compare treatment related attitudes and treatment adherence between psychotic (schizophrenia spectrum) and bipolar patients; to evaluate the relationship between treatment related attitudes, illness perceptions and health locus of control in psychotic and bipolar populations.

**Methods:**

Treatment attitude was evaluated with the Drug Attitude Scale (DAI). Treatment adherence was rated by doctors on Clinical Global Impression (CGI) Scale. Illness perceptions were evaluated with the Illness Perception Questionnaire for Schizophrenia (IPQS) and health locus of control with the Multidimensional of Health Locus of Control Scale –Form C (MHLC) at the end of inpatient care.

**Results:**

Number of participants was 51. Data indicated more positive treatment attitude in bipolar patients than in psychotic patients. MHLC scores indicated significant role in symptoms control for chance (p=0,042) and „powerful” persons (p=0,011) in psychotic patients. IPQS scores indicated that bipolar patients rather have perceptions about treatment influencing symptoms than psychotic patients. Treatment related attitudes were strongly influenced by perceptions about controllability of symptoms by treatment.

**Conclusions:**

Bipolar patients had more positive treatment attitude and perceptions about effectiveness of treatment on symptoms. This illness perception about controllability of symptoms by treatment was the strongest determinant of positive treatment attitude in this study.

**Disclosure:**

No significant relationships.

